# Sichuan paocai fermented by mixed‐starter culture of lactic acid bacteria

**DOI:** 10.1002/fsn3.1833

**Published:** 2020-08-25

**Authors:** Yuanli Luo, Yuling Liu, Ting Ren, Bin Wang, Yumei Peng, Sheng Zeng, Yu Su

**Affiliations:** ^1^ The Center of Postharvest Storage and Processing Southeast Chongqing Academy of Agricultural Sciences Chongqing China

**Keywords:** *Brassica juncea* var. *tumida* Tsen, *Lactobacillus*, *Leuconostoc*, mixed‐starter culture, Sichuan paocai, *Weissella*

## Abstract

To satisfy the demand of industrial production, selecting strains suitable for fermentation initiation is necessary. In this study, the effects of mixed‐starter culture including *Leuconostoc*, *Lactobacillus*, and *Weissella* on the quality of Sichuan pickle were discussed. Results showed that mixed‐starter culture can accelerate fermentation and had the highest efficiency for nitrite degradation, that is, the maximum nitrite concentration was 8.97 g/kg on day 1 and decreased to 1.88 mg/kg after 3 days. The mixed‐starter culture improved the sensory properties of pickles, which easily produced acids but had reduced amounts of total acids. The pickle products fermented by the mixed‐starter culture contained increased lactic acid (17.52 g/kg), mannitol (0.62%), umami (35.85), and sweet (11.36) amino acids on day 4. The strains *Weissella paramesenteroides* C2‐2 and *Lactobacillus brevis* ZP11‐2 grew well in the mixed‐starter culture fermentation.

## Graphical abstract

This is the first report about three LAB strains' application on Brassicaceae pickle. Also, the fermentation characteristics of five treatments are analyzed, including single‐starter culture fermentation, mixed‐starter culture fermentation, and spontaneous fermentation.

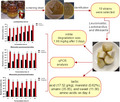

## 1 INTRODUCTION

*Brassica juncea* var*. tumida* Tsen (Brassicaceae) is a feature vegetable in Fuling, Chongqing, and is the raw material for the famous salted food “Fuling mustard” pickles. Sichuan paocai is a lactic acid‐fermented and mildly salted vegetable in China consumed as a side dish with the main meal or appetizer. The pickling method is simple. In a typical procedure, various vegetables, such as radish, cabbage, cowpea, and cucumber, are immersed in a 6%–8% salt solution containing red pepper, garlic, ginger, sugar, and other spices in a sealed jar covered with water. The fermentation is completed approximately 1 and 2 weeks during the summer and winter at ~25°C–35°C and ~8°C–15°C, respectively. In the present study, *B. juncea* var*. tumida* Tsen (Brassicaceae) was used to produce Sichuan paocai.

Homemade pickled vegetables are usually spontaneously fermented using the epiphytic microbes on the surface of the vegetables. Various microbes, for example, lactic acid bacteria (LABs) and acetic acid bacteria and yeast, are involved in fermentation, during which the variety and quantity of microorganisms constantly change (Zhao, Li, Jiang, Deng, & Kneifel, 2016). To provide uniform fermented products, selecting suitable strains to initiate fermentation is necessary. Various LABs have been used as starter culture, including *Lactobacillus brevis* (Xia et al., 2017), *Leuconostoc citreum* (Choi et al., 2003), *Enterococcus* sp. (Moon, Kang, Pyun, & Kim, 2004), *Leuconostoc mesenteroides* (Jung et al., 2012), *Bacillus coagulans* (Zhao & Ding, 2008), *Lactobacillus pentosus* (Liu et al., 2016), and *Lactobacillus plantarum* (Çon & Karasu, 2009). Compared with homofermentation, heterofermentation produces CO_2_, mannitol, and ethanol as metabolic products with less acids (Moon, Kim, & Chang, 2018). Thus, heterofermentative characteristics benefit the high sensory quality of pickle (Moon et al., 2018). Various LABs have different metabolic characteristics (Xiong, Li, Guan, Peng, & Xie, 2014) and thus produce fermentative products with different tastes and flavors. Multiple microorganisms other than a single strain used as starter culture can produce the desired quality of kimchi (Park, Seo, Kim, Byun & Son, 2019). Moreover, complex microbial consortia are more stable and have higher metabolic activity than pure culture (Smid & Lacroix, 2013). Kimchi fermentation is dominated by the genera *Leuconostoc*, *Lactobacillus*, and *Weissella* (Jung et al., 2012). *Leuconostoc*, *Lactobacillus*, and *Weissella* also play important roles in kimchi fermentation (Kang, Cho, Ahn, Lee, & Park, 2015). In the present study, the effects of mixed‐starter culture, including *Leuconostoc*, *Lactobacillus*, and *Weissella*, on the quality of Sichuan pickle were discussed.

## 2 MATERIALS AND METHODS

### 2.1 Pickle preparation

Stem lump mustard and spices were purchased from a local supermarket. The stem lump mustard was washed, naturally dried, cut into pieces, and submerged into brine solution (4% w/v NaCl) in a sealed jar (2 L) filled with water. The ratio of vegetable to cooled boiled water was 1:2. The other ingredients were as follows: sugar candy (3% w/v), ginger (4% w/v), garlic (5% w/v), hot pepper (5% w/v), and pepper (0.5% w/v). The pickle jar was filled with water to exclude air, and the fermentation was maintained at ambient temperature (20°C–25°C).

### 2.2 Isolation and identification of LABs

A total of 53 aged brine samples were collected from local farmers in Fuling, Chongqing. Pickles were prepared in accordance with the abovementioned methods with 11 aged brine instead of cooled boiled water. Brine samples were collected on days 0, 1, 2, 3, 4, 7, and 10 after vegetable submersion. The brine samples were diluted into appropriate concentrations and spread onto de Man, Rogosa, and Sharpe (MRS) agar plates containing bromcresol purple (0.4 g/L) in gas‐packed jars at 30°C for 2 days. The remaining 42 aged brine samples were directly used to isolate the LABs. The portions (100 µl) of brine samples were transferred to 900 µl of sterile water (diluted 10^–1^) and subsequently diluted to 10^–2^, 10^–3^, 10^–4^, 10^–5^, 10^–6^, and 10^–7^. When the colony number was between 30 and 300, the appropriate concentration was selected. Given the acid produced by LAB, bromocresol purple turned yellow, indicating the presence of LAB on MRS plate. The yellow colony was first selected, and the catalase‐negative, gram‐positive colony was presumed to be LAB. The heterofermentative strain that produced gas in Durham tubes was initially selected. The heterofermentative strain was then transferred to the MRS broth with 100 mg/L nitrite for nitrite degradation screening and pH measurement to perform acidification screening. The heterofermentative strain that rapidly produced acid and exhibited the highest efficiency for nitrite degradation was selected. The 16S rDNA nucleotide sequence of the strain was amplified using a Bio‐Rad T100 Thermal Cycler (Hercules, CA, USA) with the primers 27F (5′‐AGAGTTTGATCCTGGCTCAG‐3′) and 1492R (5′‐TACGGCTACCTTGTTACGACTT −3′). Each PCR was performed in a final volume of 25 µl, containing 5–10 ng of template, 2.5 µl of 10× PCR buffer, 1 µl of dNTP (2.5 mm), 1.5 µl of MgCl_2_ (25 mm), 0.5 µl of forward PCR primer (10 µM), 0.5 µl of reverse PCR primer (10 µM), and 0.15 µl of Taq DNA polymerase (5 U/µl; Takara, Japan). The PCR reaction conditions used for amplification were 95°C for 5 min, followed by 30 cycles of 95°C for 20 s, 56°C for 20 s, and 72°C for 20 s, with a final extension step of 5 min at 72°C. The PCR products were sequenced by BGI Biotechnology (Beijing, China). The obtained 16S rDNA sequences were compared with those in the GenBank database by using the BLAST search algorithm.

### 2.3 Preparation of starter culture and inoculum

Three strains, including *Weissella paramesenteroides* C2‐2 (W), *Lactobacillus brevis* ZP11‐2 (L1), and *Leuconostoc mesenteroides* 21861 (L2), were used in this study. W and L1 were isolated by the spontaneous fermentation of pickle in our laboratory. L2 was purchased from the National Research Institute of Food and Fermentation Industries of China. The two strains W and L1 preserved at low temperature and the freeze‐dried strain L2 were activated on MRS agar at 30°C for 2 days to obtain pure colony. The pure colony was then inoculated in MRS broth and cultured at 30°C for 1 day. LAB cells were harvested after centrifugation at 2,054 *g* for 10 min and washed twice with sterile 0.9% saline. The bacteria culture was plated on MRS agar at 30°C for 2 days to count the bacterial colony. According to the colony number, the bacteria culture was diluted into 10^7^ colony‐forming units (CFU)/ml with sterile 0.9% saline.

The pickle was prepared in accordance with the abovementioned methods. The treatment group was inoculated with mixed‐starter cultures at a 1:1:1 ratio of L1–L2–W. The spontaneous fermentation with no starter served as control. The inoculum size of each group was 3 ml/L pickle brine. The brine samples were collected on days 0, 1, 2, 3, 4, 7, and 10 after vegetable submersion.

### 2.4 pH and titratable acidity

Brine pH was measured using a pH meter (Leichi, Shanghai, China). Titratable acidity was determined by titrating the brine with 0.1 N NaOH and with phenolphthalein (1% m/v dissolved in 95% ethanol) as the indicator.

### 2.5 Nitrite concentration

The nitrite concentration of the brine was measured using the nitrite colorimetric method as follows: First, 5 g of brine was deproteinated and defatted by sequential precipitation with 12.5 ml of saturated sodium borate (50 g/L), 5 ml of potassium ferrocyanide (106 g/L), and 5 ml of zinc acetate (220 g/L), followed by filtration. Approximately 2 ml of sulfanilic acid (4 g/L) and 1 ml of N‐ethylenediamine dihydrochloride (2 g/L) were then added sequentially to the filtrates as color‐development reagents. The mixture was maintained at room temperature for 15 min. The absorbance (OD) value of the mixture was read at 538 nm against the reagent blank. To construct a standard curve, 0–2.5 ml of sodium nitrite solutions (200 μg/ ml) were subjected to the same color‐development process, and OD measurements were obtained.

### 2.6 Microbial analysis

The brine was diluted with 0.9% saline solution. The plate count agar (PCA) method was used to calculate the total viable counts. MRS agar (Tuopu Biol‐Engineering Co., Shangdong, China) was used for LAB counts. All plate counts were repeated thrice and incubated at 37°C for 2 days. Microbial numbers were recorded as CFU per milliliter.

### 2.7 Metabolite analysis

The brine was centrifuged at 2,054 *g* and 4°C for 10 min to harvest microorganisms. The separated supernatants and pellets were stored at −80°C for metabolite analyses and population analyses of three LABs, respectively.

Mannitol was measured using a Waters e2695 HPLC system equipped with an SPELC‐NH column (Agilent) at 35°C. The mobile phase was 78% acetonitrile and 22% water, and the flow rate was 1 ml/min. Lactic acid was analyzed using HPLC on an CAPECELL PAK MGS5 C18 column (4.6 mm × 250 mm; 5 μm) at 40°C with 0.1% phosphoric acid and methyl alcohol (97.5:2.5 volume ratio) as eluent. Acetic acid was analyzed using HPLC on an CAPECELL PAK MGS5 C18 column (4.6 mm × 250 μm; 5 μm) at 25°C with diammonium phosphate (pH adjusted to 2.7–3.5 with 1 mol/L phosphoric acid) as eluent. All samples were detected at 210 nm, except for acetic acid, which was detected at 214 nm. Amino acid content was determined using an amino acid automatic analyzer HitachI L‐8500 (Hitachi Ind., Tokyo, Japan).

### 2.8 Quantitative real‐time PCR for the 16S rRNA gene detection of three LABs

qPCR experiment was performed using the iCyclerTM Thermal Cycler system (Bio‐Rad, Hercules, CA, USA) with the designed primers (Table 1) for the three 16S rRNA gene of three LABs. Total DNA was extracted thrice from spontaneous and inoculation fermentation brine on days 0, 1, 2, 3, 4, 7, and 10 after the vegetable started fermenting. The qPCR was performed with three DNA sets at each point as templates by using SYBR^®^ Premix Ex TaqTM II (Takara, Dalian, Liaoning, China; Cat. no. RR820A). The conditions used for amplification were 95°C for 30 s, followed by 40 cycles of 95°C for 5 s, and 60°C for 34 s. The 16S rRNA gene of *Lactobacillus* was used as the internal controls to standardize transcript levels. The qPCR results were analyzed by ANOVA.

**TABLE 1:** Specific primers for three LABs designed by Primer 5 software

Primer description	Sequence
*Lactobacillus*‐F	AGCAGTAGGGAATCTTCCA
*Lactobacillus*‐R	ATTTCACCGCTACACATG
*L. brevis*‐F	ACCGTATAACAACAAAATCCGCA
*L. brevis*‐R	TCGTCTTGGTGGGCCTTTAC
*L. mesenteroides*‐F	CCTCAAGGCTGGGGATAACATT
*L. mesenteroides*‐R	ATCTCTAGGTGACGCCGAAG
W‐F	CCGTATAATACCAACAACCGCA
W‐R	GCCGTTACCTTACCAACTAGC

### 2.9 Sensory analysis

Sensory evaluations were performed by 10 panelists with experience in organoleptic evaluation. Samples were coded with random numbers. The appearance, flavor, texture, and overall acceptability were rated on a 10‐point hedonic scale (1 = dislike extremely, 5 = like moderately, and 10 = like extremely) for all samples. Water and unsalted soda crackers were provided for palate cleansing between samples.

### 2.10 Statistical analysis

Results are presented as the mean ± standard deviation for each experiment. All data were statistically analyzed by one‐way ANOVA (SPSS 17.0), and differences were considered statistically significant at *p* < .05.

## 3 RESULTS AND DISCUSSION

### 3.1 Identification of strains

A total of 154 LABs were isolated from 119 samples of paocai brine, among which 18 strains with different morphological characteristics were selected. The heterofermentative strain that produced gas in Durham tubes was selected. A total of 8 heterofermentative strains were found (Table 2). ZP11‐2 and C2‐2 had the highest efficiency for nitrite degradation with nitrite a degradation ratio of 99.16% and 98.69% at 8 hr after fermentation, respectively (Table 3). ZP11‐2 and C2‐2 also most rapidly produced acid, with a pH degradation rate of 13.34% and 12.70% at 8 hr after fermentation, respectively (Table 4). We also used *L*. *mesenteroides* due to its abundant metabolite production (Jung et al., 2012). When the three strains were mixed as one starter culture, the fermented product achieved the greatest organoleptic effects. ZP11‐2 had 99.93% sequence similarity with strain type *L. brevis* ATCC 14869^T^ (KI271266) and C2‐2 had 99.79% sequence similarity with strain type *W. paramesenteroides* ATCC 33313^T^ (ACKU01000017). According to the 16S rRNA gene‐sequence analysis, ZP11‐2 and C2‐2 were identified as *L*. *brevis* and *Weissella*, respectively.

**TABLE 2:** The heterofermentative strain screening

Strains	Produce gas	Strains	Produce gas	Strains	Produce gas
T1‐6	−	ZP1‐1	+	C1‐5	−
T2‐3	+	ZP2‐1	−	C1‐7	+
T3‐1	+	ZP4‐1	+	C2‐2	+
T4‐2	−	ZP5‐1	−	C3‐9	−
T5‐2	−	ZP11‐2	+	C4‐1	−
T6‐2	−	ZP13‐2	−	C5‐2	+

**TABLE 3:** Nitrite degradation after 48 hr

Strains	Nitrite degradation (%)	Strains	Nitrite degradation (%)
T2‐3	63.75	ZP11‐2	99.16
T3‐1	95.47	C1‐7	68.65
ZP1‐1	74.56	C2‐2	98.69
ZP4‐1	96.54	C5‐2	82.56

**TABLE 4:** Degradation rate of pH after 8 hr

Strains	Degradation rate (%)	Strains	Degradation rate (%)
T2‐3	9.95	ZP11‐2	13.34
T3‐1	11.33	C1‐7	8.65
ZP1‐1	10.96	C2‐2	12.70
ZP4‐1	10.54	C5‐2	9.16

### 3.2 pH, titratable acidity, and nitrite levels

**FIGURE 1:**
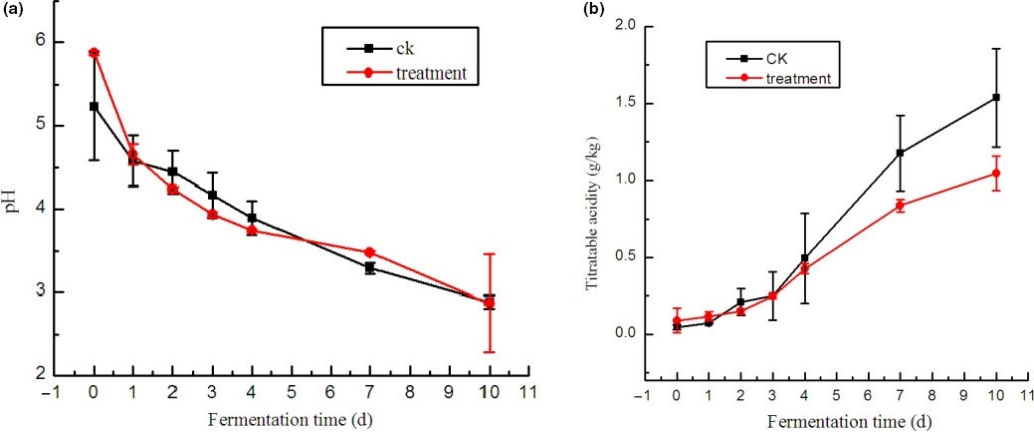
pH and titratable acidity of brine solution during fermentation. (a) pH of brine solution during fermentation. (b) Titratable acidity of the brine solution during fermentation (g/kg). The treatment group was inoculated with mixed‐starter cultures at 1:1:1 ratio of L1–L2–W. The spontaneous fermentation with no starter was as control. The brine samples were collected on days 0, 1, 2, 3, 4, 7, and 10 after vegetable submersion

As shown in Figure 1a, as fermentation proceeded, the pH decreased, and titratable acidity increased similar to previous reports (Xiong, Guan, Song, Hao, & Xie, 2012; Yan, Xue, Tan, Zhang, & Chang, 2008). In the treatment group, pH rapidly dropped from 5.87 to 3.75 after 4 days of fermentation, which was mostly approximate to the optimum fermentation. In the CK group, the pH decreased slowly from 5.24 to 3.90 after 4 days of fermentation. However, from day 4 to day 7, pH became more stable in the treatment group than that in CK, and the pH values on day 7 were 3.48 and 3.29 in the treatment and CK groups, respectively. The changes in total acidity were inversely related to the changes in pH (Figure 1b). Along with fermentation, total acidity increased in the treatment and CK groups. No evident difference was found in the total acidity of the treatment and CK groups 4 days after fermentation. On day 4, paocai was well ripened according to the total acidity (approximately 0.43) and sensory evaluation. From day 7 to day 10, the total acidity in treatment group was lower than that in CK, which may have prevented the overacidity and over‐ripening of the paocai products.

**FIGURE 2:**
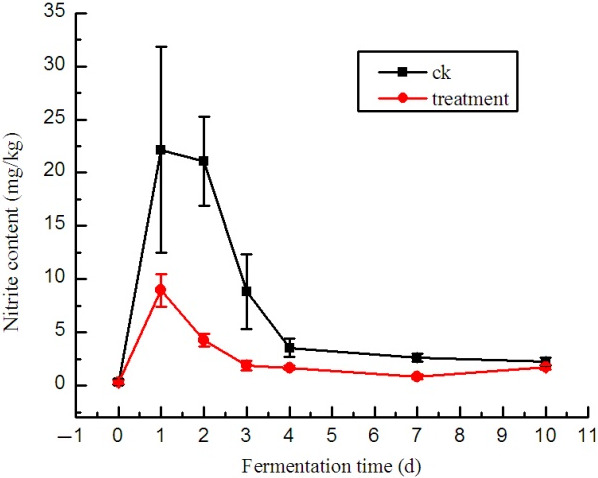
Nitrite levels of the brine solution during fermentation (mg/kg) The treatment group was inoculated with mixed‐starter cultures at 1:1:1 ratio of L1–L2–W. The spontaneous fermentation with no starter was as control. The brine samples were collected on days 0, 1, 2, 3, 4, 7, and 10 after vegetable submersion

Figure 2 shows that in the CK group, the nitrite peak appeared on day 1 during pickle fermentation, and the maximum concentration of nitrite was 22.16 mg/kg. The nitrite peak was much lower in the treatment group than that in the CK group with a maximum concentration of nitrite at 8.97 g/kg on treatment day 1. At the early fermentation stage, in the spontaneous fermentation group, LAB did not grow well to inhibit the growth of nitrate‐reducing microorganisms, which can reduce nitrate to nitrite and cause a sudden increase in nitrite concentration (Xia et al., 2017). Xia et al. (2017) reported that *L. brevis* effectively removes nitrites, and our results were consistent with theirs with a nitrite degradation ratio of 99.16% and 98.69% at 8 hr for *L. brevis* and *Weissella*, respectively. The mixed‐starter culture effectively eliminated nitrites under the joint action of the three strains.

### 3.3 Microbial analysis

**FIGURE 3:**
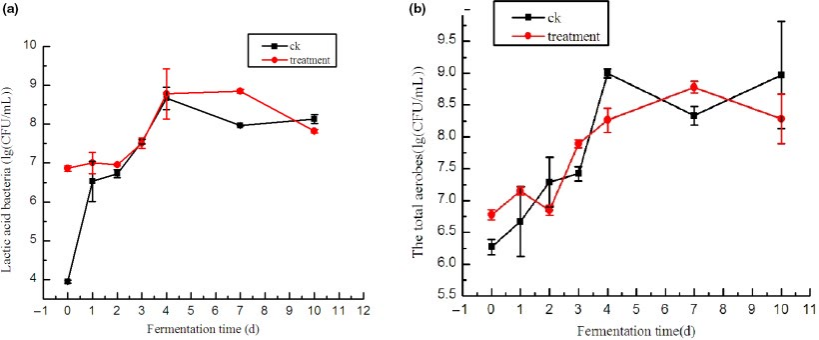
Microbial analysis in fermented paocai pickle brine. (a) Lactic acid bacteria population in fermented paocai pickle (lg(CFU/ml)). (b) Total aerobe population in fermented paocai pickle (lg(CFU/ml))

As shown in Figure 3, given that LAB can propagate on PCA plates and were the predominant microorganisms, the changes in LAB and the total aerobes showed similar tendencies. In the CK group, the CFU of LAB increased and reached the maximum on fermentation day 4. LAB decreased and then increased again from day 7 to day 10. These results were in accordance with the works of Liu et al. (2017). In the treatment group, the CFU of LAB reached the maximum on day 7 and then declined. From day 0 to day 2, the LAB population in the treatment group was higher than that in the CK group. The high population of LAB benefits the fermentation and control of pathogenic and spoilage microorganisms (Gao, Li, & Liu, 2014). The LAB can antagonize nitrate‐reducing microorganisms and deplete nitrite (Yan et al., 2008), so only small nitrite fluctuations occur in cabbage inoculated with starter cultures (Yan et al., 2008). Our results were consistent with theirs, and the nitrite peak was much lower in the treatment group than that in the CK group. The addition of mixed‐starter culture may change the microbial flora in paocai brine. Thus, difference was observed in the total aerobe distribution in the treatment and CK groups.

### 3.4 Metabolic product analysis

Organic acids, such as lactic and acetic acids, and flavoring compounds, such as mannitol and amino acids are related to pickle tastes and flavors (Jung et al., 2012). As shown in Table 5, the amount of lactic acid produced by inoculated starter culture was much higher than that produced by spontaneous fermentation. In the spontaneous fermentation, very few LABs were found in brine at the early fermentation stage. The CFUs of LAB were 3.95 and 6.60–6.86 in spontaneous and inoculated fermentation on day 0, respectively. The amount of lactic acid might be related with the growth of the LAB population. Compared with the CK group, the treatment group showed higher mannitol content and lower acetic acid amount. Mannitol is a natural six‐carbon diabetic polyol with refreshing taste and is produced by consuming fructose (Grobben et al., 2001). Mannitol production may improve kimchi taste and flavor (Otgonbayar, Eom, Kim, Ko, & Han, 2011). A moderate amount of acetic acid can improve the stability and organoleptic quality of food (Oliveira, Perego, Oliveira, & Converti, 2012). In our study, the increased production of mannitol and a slight amount of acetic acid might have acted together to improve the taste of paocai pickle for mixed culture. As illustrated in Table 6, compared with CK, the treatment group exhibited higher umami and sweet amino acid total sum values and lower bitter amino acid amount. The umami and sweet amino acids might be beneficial for the organoleptic quality of paocai.

**TABLE 5:** The metabolite product in the fermented Paocai pickle brine on the 4th day

Item	Different treatment groups	
	CK	Treatment
Mannitol (%)	0.48 ± 0.07	0.62 ± 0.07
Acetic acid (g/kg)	0.44 ± 0.05	0.19 ± 0.03
Lactic acid (g/kg)	5.76 ± 0.06	17.52 ± 0.12

**TABLE 6:** The amino acid production in the fermented Paocai pickle brine on the 4th day (mg/100 ml)

Item	Different treatment groups		
	Amino acid	CK	Treatment
Umami	Asp	0.5	31.43
	Glu	4	4.42
	Umami sum	4.5	35.85
Sweet	Ala	1.93	2.99
	Gly	0.69	0.46
	Ser	0.93	1.82
	Thr	3.5	6.09
	Pro	–	–
	Sweet sum	7.05	11.36
Bitter	Arg	0.03	0.06
	His	–	2.69
	Ile	1.96	1.16
	Leu	1.03	0.59
	Met	0.03	0.08
	Phe	2.42	1.07
	Tyr	1.37	0.25
	Lys	2.32	1.11
	Val	5.61	3.06
	Bitter sum	14.77	10.07

### 3.5 Sensory analysis

As shown in Table 7, the appearance, flavor, texture, and overall acceptability were all better in the treatment group than that in the CK group. This result was consistent with the physicochemical characteristics and metabolic product analysis. Therefore, the application of mixed‐starter culture can improve the quality of paocai pickle.

**TABLE 7:** Sensory analysis of the fermented Paocai pickle

Item	CK	Treatment
Appearance	5.5 ± 1.08	6.1 ± 1.45
Flavor	5.2 ± 1.40	6.9 ± 1.37
Texture	4.5 ± 1.27	6.1 ± 1.20
Overall acceptability	15.2 ± 2.35	19.1 ± 2.42

### 3.6 qPCR analysis of changes in the three LABs during fermentation

**FIGURE 4:**
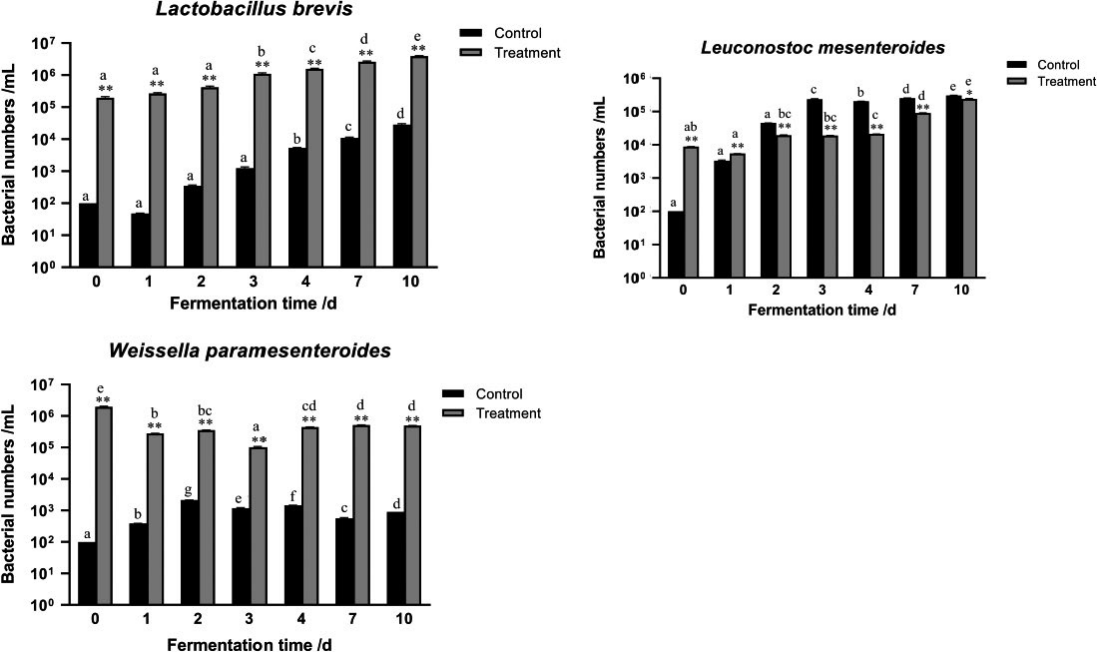
qPCR analysis of three LABs in paocai brine. Asterisks indicate significant differences (*Pb0.05 and **Pb0.01) between treatment and control. A letter indicates differences compared with that on 0 day in the same treatment. Error bars represent the mean ± *SD* of three independent PCR amplifications and quantifications

As shown in Figure 4, the population of *L. brevis* increased in both spontaneous and mixed‐culture fermentation, and the number of *L. brevis* was much higher in the mixed‐culture fermentation than that in spontaneous fermentation. This finding indicated that *L. brevis* was acid tolerant and could grow well in the mixed culture. In spontaneous fermentation, the number of *Leuconostoc* increased and reached the highest value on day 3 and then remained stable. *L*. *mesenteroides* are poorly acid resistant (Xiong et al., 2014). Thus, we speculated that the population of *L*. *mesenteroides* decreased at the late fermentation stage. RT‐PCR detected viable and dead bacteria, so the number of *Leuconostoc* remained stable in our study. The population of *L*. *mesenteroides* in mixed‐culture fermentation was higher than that in spontaneous fermentation only on day 0, suggesting that *L*. *mesenteroides* were poorly acid resistant and that mixed‐culture fermentation produced acid earlier and more abundantly than that in spontaneous fermentation (Table 1). The population of *Weissella* in the mixed‐culture fermentation was higher than those in the spontaneous fermentation throughout the entire fermentation process, suggesting that *Weissella* can grow well in mixed culture.

## 4 CONCLUSION

Mixed‐starter cultures, including the three LABs, were applied to paocai fermentation, which enabled the use of brine with low salt concentration and fermentation acceleration. Compared with spontaneous fermentation, the mixed‐starter culture yielded pickles with better sensory properties, indicating its high potential as an alternative to age brine for Sichuan paocai preparation. Mixed‐starter culture produced acid early, but the amount of total acid was low, which can strengthen the organoleptic quality due to the reduced amount of produced acid (Choi et al., 2003). Mixed‐starter culture exhibited high efficiency for nitrite degradation and promoted the growth of LAB at the early fermentation stage. The pickle product fermented by mixed‐starter culture contained increased lactic acid, mannitol, and umami and sweet amino acids and improved organoleptic evaluation. The strains W and L1 can grow well in the mixed‐starter culture fermentation. L2 existed primarily at the early fermentation stage, and its metabolic product may act on the quality of paocai pickle.

## CONFLICT OF INTERESTS

The authors hereby declare no potential conflicts of interest.
